# A MetAP2 inhibitor blocks adipogenesis, yet improves glucose uptake in cells

**DOI:** 10.1080/21623945.2019.1636627

**Published:** 2019-07-02

**Authors:** Md Abu Bakkar Siddik, Bhaskar C. Das, Louis Weiss, Nikhil V. Dhurandhar, Vijay Hegde

**Affiliations:** a Department of Nutritional Sciences, Texas Tech University, Lubbock, TX, USA; b The Icahn School of Medicine, Department of Medicine, New York, NY, USA; c Department of Pathology, The Albert Einstein College of Medicine, New York, NY, USA

**Keywords:** Angiogenesis, adipogenesis, lipid accumulation, glucose uptake, MetAP2 inhibitor, fumagillin

## Abstract

Adipose tissue expansion involves angiogenesis to remodel its capillary network. The enzymemethionine aminopeptidase 2(MetAP2) promotes angiogenesis.MetAP2 inhibitors suppress angiogenesis and have potential anti-obesity effect. However, impairment in adipose tissue expansion is also linked with impaired glycemic control.This study investigated the effect of BL6, a MetAP2 inhibitor, on adipogenesis and glucose disposal.To test effect on angiogenesis, Human Umbilical Vein Endothelial Cells(HUVECs) were treated with BL6 for 24h to determine tube formation. Further, to test effect on adipogenesis and glucose disposal,3T3-L1 pre-adipocytes were treated with BL6(0 µM, 20µM, 50 µM or 100µM) during differentiation. Differentiated cells were stained with Oil Red O for determining lipid accumulation, and glucose uptake assay. Protein levels and RNA expression for key genes involved in the adipogenic cascade were determined.BL6 treatment of HUVECs dose dependently blocked angiogenesis. During differentiation of pre-adipocytes, 50μM and 100µM BL6 significantly reduced lipid accumulation. Treatment with 100µM BL6 significantly decreased expression of adipogenic genes. Interestingly, BL6 treatment dose dependently increased glucose uptake by 3T3-L1 cells.MetAP2 inhibitor blocks angiogenesis, attenuates adipogenesis, yet increases cellular glucose uptake. Collectively this proof of concept study supports a possible role for MetAP2 inhibitor BL6, as a putative anti-obesity therapeutic agent.

## Introduction

Obesity is highly prevalent in many developed countries and its prevalence is increasing worldwide[1]. Obesity, characterized by excess accumulation of adipose tissue, is a complex metabolic disorder that is commonly associated with type-2 diabetes mellitus (T2D), hypertension, coronary heart disease, stroke, dyslipidemia, gallbladder disease, hepatic steatosis, sleep apnea, stroke, endometrial disorder, and cancer [1–3]. Diet and exercise remain the cornerstones of obesity management; however, it is likely that many patients will require anti-obesity drugs to reduce body weight and prevent complications [4]. Adipose tissue is highly vascularized in which the adipocytes are nourished by an extensive capillary network [5–7]. Expansion of adipogenesis requires angiogenesis [5], whereas, inadequate angiogenesis or vascular dysfunction is closely associated with many metabolic disorders [8–10].

Methionine amino peptidase 2 (MetAP2), a metalloproteinase, is a regulatory element for angiogenesis and a target molecule for anti-angiogenic compounds [11–13]. In pre-clinical models of obesity and diabetes, MetAP2 inhibitors have been shown to inhibit fat mass expansion and improve glycemic control, and reduce food intake in mice [14–16]. These findings have paved avenues for possible therapeutic intervention of obesity and obesity-associated disorders by targeting the vascular compartment. In clinical studies of obesity, beloranib, a MetAP2 inhibitor substantially increased weight loss along with improved glycemic control [17–20]. However, in spite of these promising findings, a phase 2 clinical trial testing the long-term effects of beloranib had to be stopped early because of an imbalance of venous thromboembolism along with other adverse effects in beloranib treated individuals [21].

Collectively, these significant efficacy findings support further investigation of other MetAP2 inhibitors that might alleviate some of these risks and adverse effects previously observed. Here we report the synthesis and characterization of a novel MetAP2 inhibitor compound BL6.

Fumagillin is a natural product first isolated from *Aspergillus fumigatus* in 1949, whereas its molecular target, MetAP2, was only identified in 1997 [22]. It acts by covalently modifying His231 in the active site of MetAP2 and does not inhibit MetAP1 [22,23]. In early clinical studies, it was tested primarily for oncologic indications and as an anti-parasitic agent; it was well tolerated and associated with few adverse effects [17–19]. Later, a new derivative of fumagillin was synthesized, the fumagillin-like synthetic compound TNP-470 (AGM-1470) [14,16].

We have reshaped the chemical structure of fumagillin (Supplemental Figure 1A) to generate a boron atom containing MetAP2 inhibitor analog BL6, which is unique in nature and different in chemical structure with a different pharmacophore group (Supplemental Figure 1B). In this pilot study, we investigated the effect of BL6 on angiogenesis by measuring block to tube formation in human umbilical vein endothelial cells (HUVECs). We further examined its anti-adipogenic effect by determining the inhibition of lipid accumulation in pre-adipocytes during differentiation. Dose-dependent block to adipogenesis was confirmed by gene and protein expression. Finally, the effect of a block to adipogenesis on glucose metabolism was determined by glucose uptake assay in pre-adipocytes treated with compound BL6 during adipogenesis.

## Material and methods

Experimental outlines are described below. Details of assays are presented under ‘Techniques and Assays’ (T&A) section.

### Experiment 1: does BL6 block angiogenesis?

HUVECs (cell applications, Catalog No. 200-05n), 20,000 to 30,000 were seeded per well of a 96-well plate on matrigel (Corning, Catalog No. 356,234) to promote tube formation. Three wells of cells were seeded per group for five groups of cells; control (no treatment), control DMSO (dimethyl sulfoxide), or 20 µM, 50 µM or 100 µM of BL6 in DMSO. After 24 h, each well was visualized for tube formation. Images were taken and tube length measured using the NIH image j software to determine block to angiogenesis.

### Experiment 2: does BL6 block adipogenesis?

Murine 3T3-L1 (passage 3, ATCC Catalog No. CL-173) pre-adipocyte cells were treated with increasing doses of BL6 (0 µM, 20 µM, 50 µM and 100 µM with respective DMSO controls adjusted for volume) during the adipogenesis process induced with differentiation media. Two millilitres of media, either containing BL6 or respective volume of DMSO, was added to each well. The corresponding volume of DMSO 2 μl, 5 μl, and 10 μl was added per ml for treatment with 20 μM, 50 μM, and 100 μM of BL6, respectively. Following differentiation for 8 days with BL6 refreshed during media change every 2 days, cells were stained with Oil Red O dye and the dye was extracted to quantify lipid accumulation as described in T&A.

### Experiment 3: Does BL6 block gene expression and molecular signalling of proteins in the adipogenic pathway?

In a parallel experiment as described in experiment 2, protein lysates from 3T3-L1 pre-adipocyte cells treated with BL6 (0 µM, 20 µM, 50 µM and 100 µM with respective DMSO controls adjusted for volume) were separated on a SDS-PAGE gel and immunoblotted for Adiponectin, peroxisome proliferator-activated receptor gamma (PPARγ), C/EBPα, C/EBP β, fatty acid synthase (FAS), pAKT, and glucose transporter 4 (Glut4) proteins, which were normalized to glyceraldehyde 3-phosphate dehydrogenase (GAPDH), α-tubulin or β-actin by western blotting. Another set of similarly treated cells were lysed following 8 days of differentiation to extract RNA to determine gene expression as described in T&A. Gene expressions of Adiponectin, PPARγ, SREBP1c, C/EBPα, C/EBP β and FAS were measured and normalized to that of GAPDH﻿.

### Experiment 4: BL6 enhances cellular glucose uptake independent of a block to adipogenesis

Glucose metabolism was determined following block to adipogenesis by treating 3T3-L1 cells with 0 µM, 20 µM, or 100 µM BL6 in DMSO. Cells were treated with adipogenesis inducing media as described in experiment 2. Following cell differentiation for 8 days, glucose uptake assay was conducted under basal and insulin-stimulated conditions as described in T & A.

Each experiment was repeated a minimum of 4–5 times.

## Techniques and assay

### Cell culture

#### 3T3-L1 pre-adipocyte media

Ten percent Hyclone Bovine Calf Serum Defined Iron Supplemented (Hyclone, Catalog No.SH30072.03) was used with 500 mL DMEM cells media (Cellgro, Catalog No. 10–017-CV) along with 1% antibiotic (containing penicillin and streptomycin antibiotic-antimycotic solution; SIGMA-Aldrich, Catalog No. A5955)

#### 3T3-L1 adipocytes media

Ten percent Hyclone Fetal Bovine Serum-Characterized (Hyclone Catalog No. SH30071.03) was used with 500 mL DMEM cell media (Cellgro Catalog No. 10–017-CV) with 1% antibiotic (containing penicillin and streptomycin antibiotic-antimycotic solution; SIGMA-Aldrich, Catalog No. A5955).

## Induction of adipocyte differentiation

The adipocyte differentiation medium +100 nM human insulin +1 *μ*M dexamethasone (Alfa Aesar, Catalog No. A17590) +250 *μ*M 3-isobutyl-1-methylxanthine [IBMX](Sigma-Aldrich Catalog No. I5879) was added to 3T3-L1 cells two days post confluency. Two days later, the medium was changed to adipocyte media +0.250 nM insulin and replaced every 2 days thereafter for 8 days.

Reagents were as follows: Insulin stock solution (1 mg/ml) (Sigma-Aldrich Catalog No. 10,516), dexamethasone stock solution (3.9 mg/mL)(Alfa Aesar, Catalog # A17590), methylisobutylxanthine (3-isobutyl-1-methylxanthine) (Sigma-Aldrich Catalog No. I5879).

## Determination of lipid accumulation

Oil red O, a lipid-specific dye, was used to determine lipid accumulation in murine 3T3-L1 adipocytes as described [24]. Cells were fixed for 1 h with 10% formalin solution (Sigma-Aldrich; catalog no. HT551128), washed with water, and stained for 2 h with oil red O (EMD bioscience, San Diego, CA, USA Catalog No. 3125–12), followed by 2–3 times washing with water. The dye was extracted with isopropyl alcohol (Fisher Chemical, Catalog No. A464-4), and its absorbance was read at 510 nm.

## Synthesis of the compound BL6

### Synthesis

Compound **5** (Supplemental Figure 1B) (0.28 g, 1.00 mmol, 1.00 equivalent) was dissolved in dry dimethylformamide (DMF) (2 mL) and CH_2_Cl_2_ (4 mL). After stirring for 1 h, oxalyl chloride (COCl)_2_ was added slowly at 0°C under N_2_ gas. The mixture was stirred at room temperature for 1.5 h. Dichloromethane was removed carefully and dry DMF (3 mL) was added for immediate use. The DMF solution was then added to the stirred mixture of 4-aminophenyl boronic acid pinacol ester [25], triethylamine (Et_3_N) and DMF (2 mL) slowly at 0°C under N_2_ gas. The reaction mixture was stirred at room temperature for 3 h under N_2_ gas. It was quenched with saturated NH_4_Cl and extracted with EtOAc (40 mL). The organic phase was washed with H_2_O (15 mL × 3), satd. NaCl (10 mL), dried over Na_2_SO_4_ and filtered. The organic layer was concentrated to get a yellow solid.

### Purification

It was further purified by silica gel column chromatography using ethyl acetate and hexane as eluents to get 0.112 g of compound BL#6 as white solid (23.0%, 0.23 mmol) (Supplemental Figure 1(b)). Rf (ethyl acetate/n-hexane 1: 5 v/v): 0.30.

### Analytical data-structure elucidation

After purification, we elucidated the structure using NMR (Nuclaer Magnetic Resonance). We matched the observed value of Proton ^1^H and carbon ^13^C of our BL6 compound with theoretical value. These are a routine test with organic chemistry to identify the newly synthesized compounds.

^1^H NMR (400 MHz, DMSO-d_6_) *δ* 10.30 (s, 1H, -NH-), 7.93 (d, *J* = 8.41 Hz, 2H), 7.82 (d, *J* = 8.59 Hz, 2H), 7.65 (d, *J* = 8.54 Hz, 2H), 7.43 (d, *J* = 8.34 Hz, 2H), 6.49 (s, 1H), 2.58 (t, *J* = 6.24 Hz, 2H), 2.20 (q, *J* = 7.43 Hz, 2H), 1.88 (s, 3H), 1.46 (t, *J* = 6.24 Hz, 2H), 1.29 (s, 12H), 1.06 (s, 6H), 1.03 (t, *J* = 7.43 Hz, 3H). ^13^C NMR (400 MHz, DMSO-d_6_) *δ* 165.25, 147.97, 142.02, 141.68, 140.89, 134.99, 131.68, 128.83, 127.39, 126.53, 120.39, 119.10, 83.35, 38.10, 35.39, 27.29, 24.58, 22.26, 14.82, 14.54.

## Immunoblotting

Cultured cells were lysed in radioimmunoprecipitation assay buffer (RIPA) buffer (#sc-24,948; SantaCruz Biotechnology) with added protein inhibitor cocktail. Protein from the lysates was collected after centrifugation (13,000 G, 4°C, 15 min) and measured by bicinchoninic acid protein assay (#B9643, #C2284; Sigma-Aldrich). 30 μg lysates were subjected to SDS-PAGE (7.5 or 15% polyacrylamide), and transferred to polyvinylidene difluoride membranes (#162–0177; Bio-rad), incubated with primary antibody [PPARγ, SantaCruz Sc-7273 (1:500), C/EBP α, SantaCruz Sc-365,318 (1:500), C/EBP β, SantaCruz Sc-7962 (1:500), GAPDH, Cell signaling, Danvers, MA, USA 2118s (1:10,000) Adiponectin, Millipore MAB3832 (1:500), PPARγ, SantaCruz Sc-7273 (1:500), C/EBP α, SantaCruz Sc-365,318 (1:500), C/EBP β, SantaCruz Sc-7962 (1:500), GAPDH, Cell signaling 2118s (1:10,000) Adiponectin, Millipore, MAB3832 (1:5000)], and then quantified by secondary antibody conjugated with horseradish peroxidase and ECL detection reagents (Amersham Biosciences, #RPN2209). Protein expression was normalized to GAPDH and total AKT, and measured by densitometry using Image J software (National Institutes of Health).

## RNA extraction and gene expression analysis

Cells grown as a monolayer in six well plates were treated with BL6 along with MDI for 8 days of differentiation period. Cells were homogenized by using 990 µl of QIAzol Lysis Reagent (Qiagen, Cat. No.79,306) to each well of six well plates. Total RNA was extracted using RNeasy Plus Universal Mini Kit (Qiagen) and measured by using CYTATION 3 imaging reader (BioTek, Winooski, VT). cDNA was generated from 1 µg total RNA by using iScriptTM Reverse Transcription Supermix (Biorad, Cat.# 1,708,841). The following reaction protocols were used (Mastercycler, Eppendorf): 25°C for 5 min, 46°C for 20 min, 95°C for 1 min. The cDNA was diluted 1:10 with nuclease-free water and stored at −20. cDNA (at a final concentration of 10ng/µL) was amplified using iTaq Universal SYBR green supermix (Biorad,) on a CFX-Connect real-time PCR instrument (Biorad). Amplification protocol for all genes was as follows: Initial denaturing at 95°C for 2 min, 40 cycles of denaturation at 95°C for 5 s, annealing at 60°C for 30 s and completed with the following steps – 95°C for 5 s, 65°C for 5 s and 95°C for 5 s. Ct values were determined and final relative quantification was determined using the delta-delta Ct method with GAPDH and β2M as the house-keeping gene.

## Primers used for gene expression

**PPARγ-F**- TGGAATTAGATGACAGTGACTTGG; **PPARγ-R**- GAGCACCTTGGCGAACAG, **Adiponectin-F**- GCTCTCCTGTTCCTCTTAATCCT; **Adiponectin-R**- AGTGCCATCTCTGCCATCA, **C/EBP α**-F- AGTAACCTTGTGCCTTGGA; **C/EBP α**-R- GCTTCCTGTATCTTCCTCCT, **MetAP2-F**- AGATACGACAGATAACCTCAGATT; **MetAP2-R**- AACCTTCCTCAACTATACTCCTT, **C/EBP β-F**- CTGAGCGACGAGTACAAGAT; **C/EBP β-R**- GCTGCTCCACCTTCTTCT, **Glut 4-F**- CCAGCCTACGCCACCATA; **Glut 4-R**- GTTCCAGCAGCAGCAGAG, **FASn-F**- TGGCTCACAGTTAAGAGTTCA; **FASn-R**- GCCTCCTTGATATAATCCTTCTG, **SREBP 1-F**- GCTTCTCTTCTGCTTCTCTG; **SREBP 1-R**- GGCTGTAGGATGGTGAGT, **GAPDH-F**- GGTGAAGGTCGGTGTGAAC; **GAPDH-R**- TGAGTGGAGTCATACTGGAACA.

## Glucose uptake

As previously described [26,27], 3T3-L1 cells, treated with BL6 for 8 days during the differentiation period as described in experiment 2, were exposed to serum-free media for 2 h, then washed twice with PBS, followed by 112.5 μL Krebs-Ringer phosphate buffer treatment (136 mM NaCl, 4.7 mM KCl, 10 mM NaPO_4_, 0.9 mM CaCl_2_, 0.9mM MgSO_4_). To determine non-specific glucose uptake, one well of cells was treated with100 nM cytochalasin B (#6762, Sigma Aldrich). Next, 12.5 μL of 10X isotope solution was added to each well to a final concentration of 100 nM 2-deoxyglucose and 0.5 μCi ml-1 [3H]-2-deoxyglucose (#NEC720A250UC, PerkinElmer) for 5 min. Cells were immediately washed in ice-cold PBS. Next, 500 μL of 0.05% SDS was added to each well, and incubated at 37°C for 30 min. 450 μL of cell lysate from each well was added to individual scintillation vials. The remaining 50 μL cell lysate was used for protein determination with BCA assay. The scintillation counts per minute were normalized to the protein content of each well.

## Statistics

All values are expressed as mean ± standard deviation (SD). The glucose uptake and signalling experiments were repeated three times or more, and similar results were obtained. Statistical differences between two group means were determined by Student’s *t* test, and the differences were considered significant at *p* < 0.05. One-way ANOVA was used for multiple comparisons followed by tukey’s test. Groups not sharing a letter denote statistical significance. Significance was considered at *p* < 0.05.

## Results

### Experiment 1: BL6 blocks angiogenesis

**Figure 1.**
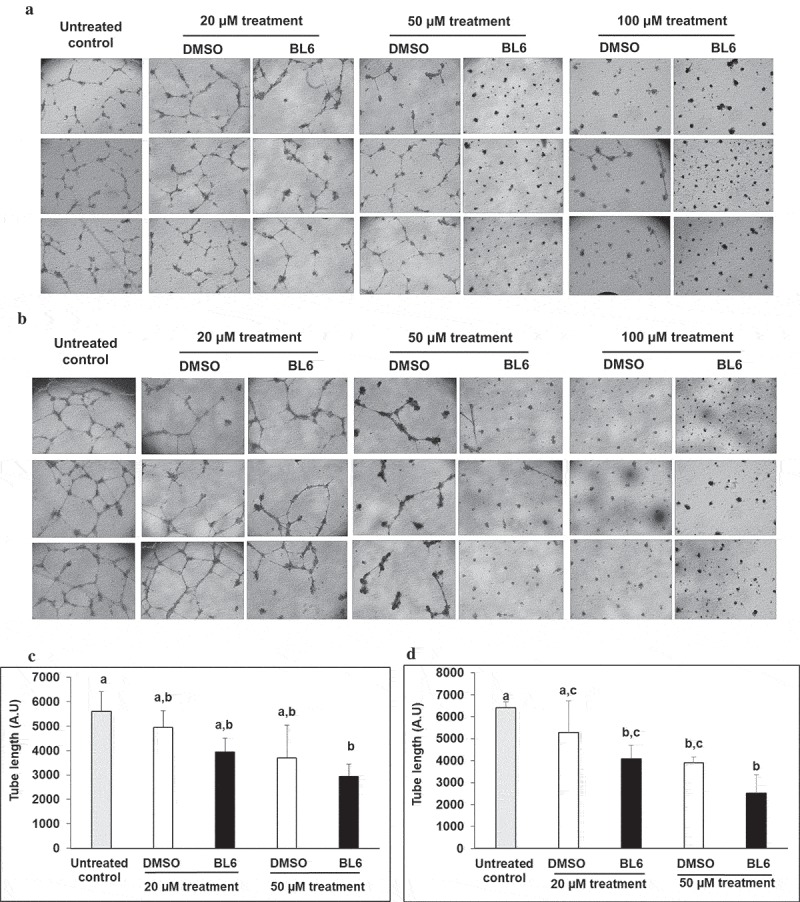
Block to angiogenesis measured by tube formation in HUVECs. 20,000 (a) and 30,000 (b) cells were treated with 20 µM, 50 µM and 100 µM of BL6 and their respective tube length measured (c, d). Cells were cultured for 24 hours on Matrigel at 37°C and tube formation was determined following 24 hours. The effect of each dose of BL6 was compared to its own DMSO control. The experiment was repeated a minimum of three times.

To determine the ability of MetAP2 inhibitor compound BL6 to block angiogenesis, 20,000 or 30,000 HUVECs were treated with increasing doses of the compound. We first tested 0 µM, 100 µM, 200 µM, and 1 mM compound BL6 on 20,000 HUVECs and found 200 µM and 1 mM to be toxic to cells (data not shown). Therefore, we used 0 µM, 20 µM, 50 µM, and 100 µM for subsequent testing. HUVECs show visual disruption in tube formation with 20 µM and 50 µM of BL6 (Figure 1(a,b)). There appears to be some block to tube formation by DMSO alone when compared with no DMSO control cells (Figure 1(a,b)). Therefore, cells treated with different concentrations of BL6 were compared to their respective DMSO controls adjusted for volume. However, despite the DMSO effect, inhibition of tube formation by different concentrations of BL6 is visually evident. Treatment with 100uM BL6 did not show any visually observable tube formation; therefore, tube formations in these cells were not quantified (Figure 1(a,b)).

To quantify tube formation, tube length was measured using the Image j software. Both 20,000 and 30,000 HUVECs show a significant reduction in total tube length when treated with 20 µM, and 50 µM of the anti-angiogenic compound BL6 (Figure 1(c,d)).

### Experiment 2: BL6 blocks adipogenesis

**Figure 2.**
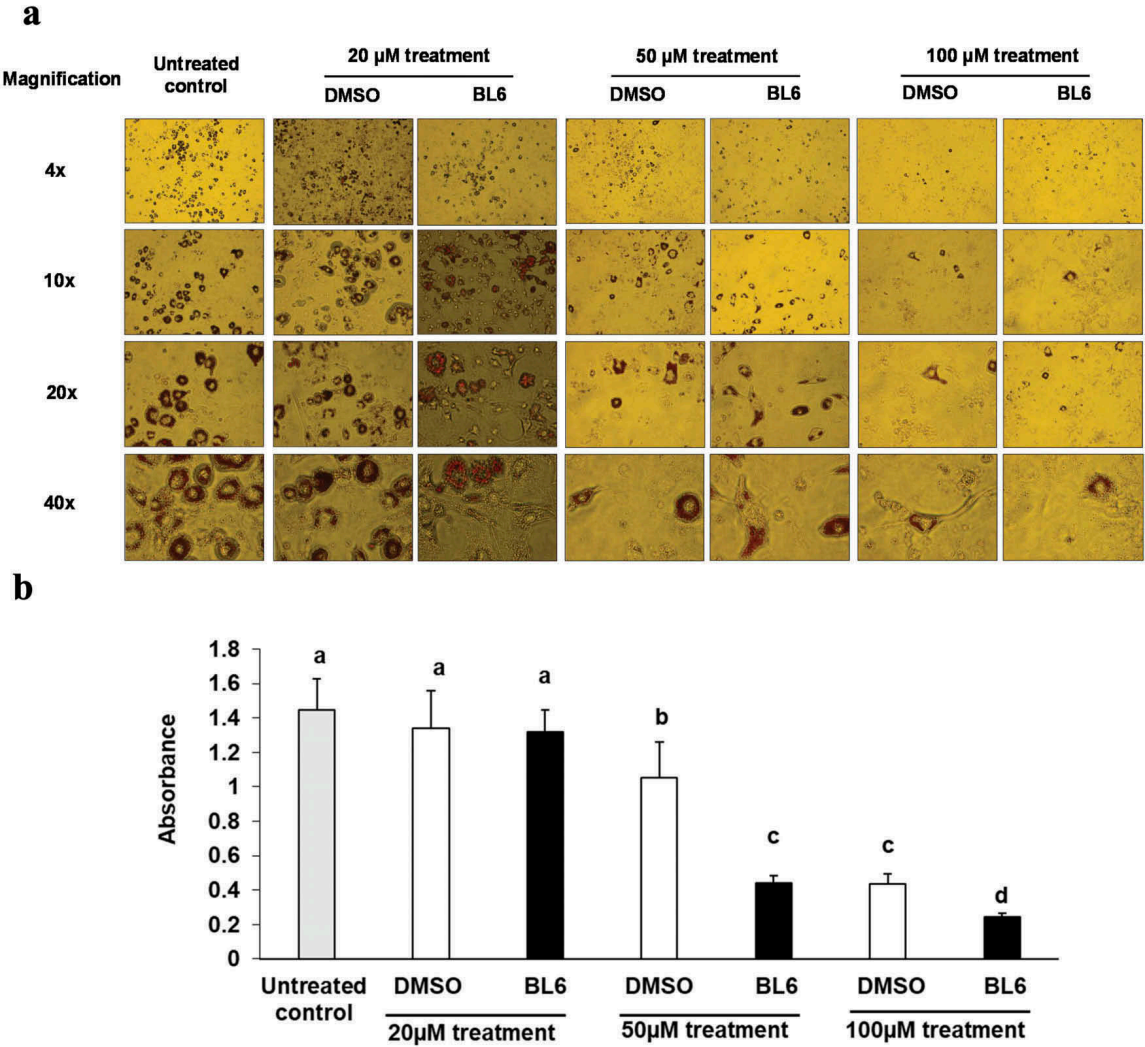
Effect of BL6 on adipocyte differentiation (a) and corresponding Oil Red O staining (b) in cultured 3T3-L1 adipocytes. (a) 3T3-L1 pre-adipocyte cells were treated with 20 µM, 50 µM and 100 µM of BL6 along with MDI for 8 days during differentiation. Block to adipogenesis was visually observed following oil red O staining for lipid. (b) Lipid staining was quantified by measuring absorbance. Y-axis shows absorbance of Oil Red O dye at 510 ηm. Data are presented as average absorbance ± sd (n = 6). The effect of each dose of BL6 was compared to its own DMSO control. Groups sharing different alphabet denote statistical significance (P < 0.05). The experiment was repeated a minimum of three times.

To determine if dose-dependent block to angiogenesis by BL6 also blocks adipogenesis and lipid accumulation, 3T3-L1 cells were treated with increasing doses of BL6 (0 µM, 20 µM, 50 µM and100 µM of BL6 along with corresponding DMSO control adjusted for volume) during differentiation with MDI cocktail (Figure 2(a)). Following differentiation for 8 days, cells were stained with oil red O dye and the dye was extracted to quantify lipid accumulation. In a dose-dependent manner, BL6 significantly blocks adipogenesis as indicated by lipid accumulation (Figure 2(b)). To determine if compound BL6 can block adipogenesis and lipid accumulation after differentiation is induced, we treated 3T3-L1 cells with BL6, 2 days following adipogenesis induction with MDI cocktail. We observed block to adipogenesis and lower lipid accumulation (20 µM p = 0.06; 100 µM p < 0.005) (data not shown). Changes in gene and protein expression with these observations will be determined in future studies. In the current study further analysis was done treating cells with compound BL6 along with MDI cocktail during differentiation.

### Experiment 3: (a) BL6 down-regulates the expression of adipogenic genes

To determine changes in gene expression by BL6 treatment, quantitative real-time polymerase chain reaction (RT-PCR) was performed. RNA extracted from 3T3-L1 cells treated with BL6 (0 µM, 20 µM, 50 µM and 100 µM) during differentiation was used to determine the expression of MetAP2, PPARγ, FAS, adiponectin, SREBP1, C/EBP-β and C/EBPα. We first determined gene expression of the MetAP2 gene in these cells following BL6 treatment, which is down-regulated in a dose-dependent manner as expected (Figure 3). Treatment with 100 μM of BL6 shows significantly reduced expression of FAS, SREBP1, and C/EBP α compared with DMSO treated cells (Figure 3(c)). Furthermore, PPARγ, C/EBP β and adiponectin involved in the adipogenic pathway were also lower by approximately 30% further confirming block to adipogenesis by MetAP2 inhibitor BL6, but the difference was not statistically significantly different.

**Figure 3.**
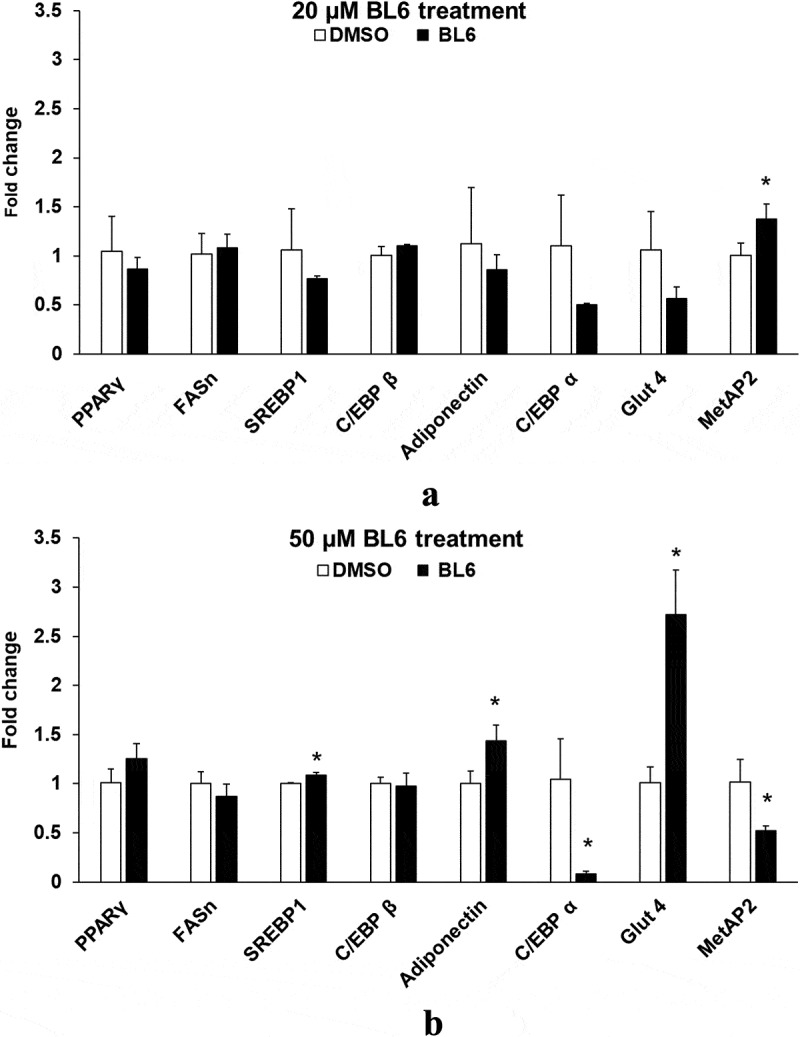
Gene expression of PPARγ, FASn, SREBP1, C/EBP β, adiponectin, C/EBP α, Glut4 and MetAP2 for 20 µM (a), 50 µM (b) and 100 µM (c) of BL6 treatment along with their respective DMSO control. Real time qRT-PCR was performed to determine the relative gene expression following BL6 treatment. The Y-axis shows fold-change difference in expression. The effect for each dose of BL6 was compared to its own DMSO control. Data are presented as average fold change ± sd (n = 3). Bars showing asterisks on top denote statistical significance (P < 0.05). The experiment was repeated a minimum of three times.

**Figure 3.**
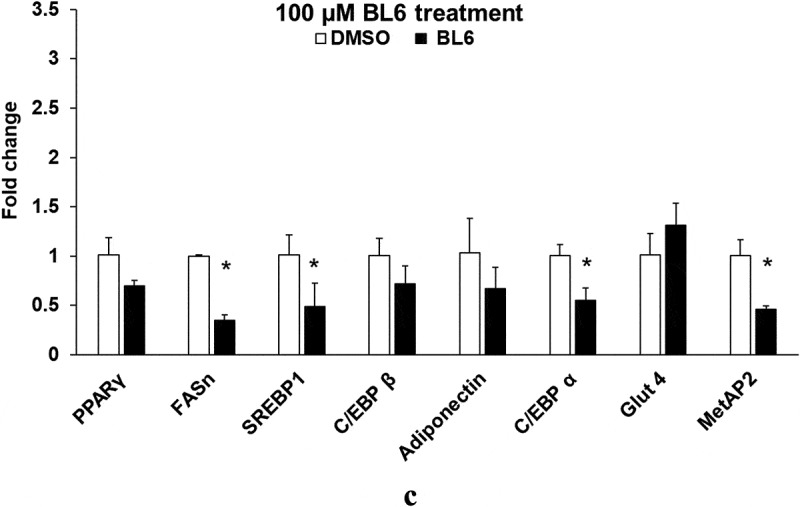
Continued

### (b) protein expression of adipogenic genes increased following treatment with BL6 compared with untreated control

Cellular protein lysates were extracted from 3T3-L1 cells treated with BL6 (0 µM, 20 µM, 50 µM and 100 µM) for 8 days during differentiation to determine changes in molecular signalling by western blotting analysis. Genes involved in the adipogenic pathway (Figures 4 and 5), PPARγ (A), C/EBP α (B), FAS (C), and adiponectin (D) all showed significantly increased protein expression despite block to adipogenesis and lipid accumulation. Immunoblotting for genes playing a role in cellular glucose uptake showed a dose-dependent increase in pAKT expression with 20 µM and 50 µM compound BL6 treatment (Figure 4(e)) and a highly significant increase in Glut4 abundance for all three doses of MetAP2 inhibitor treatment (Figures 4 and 5(f)).

**Figure 4.**
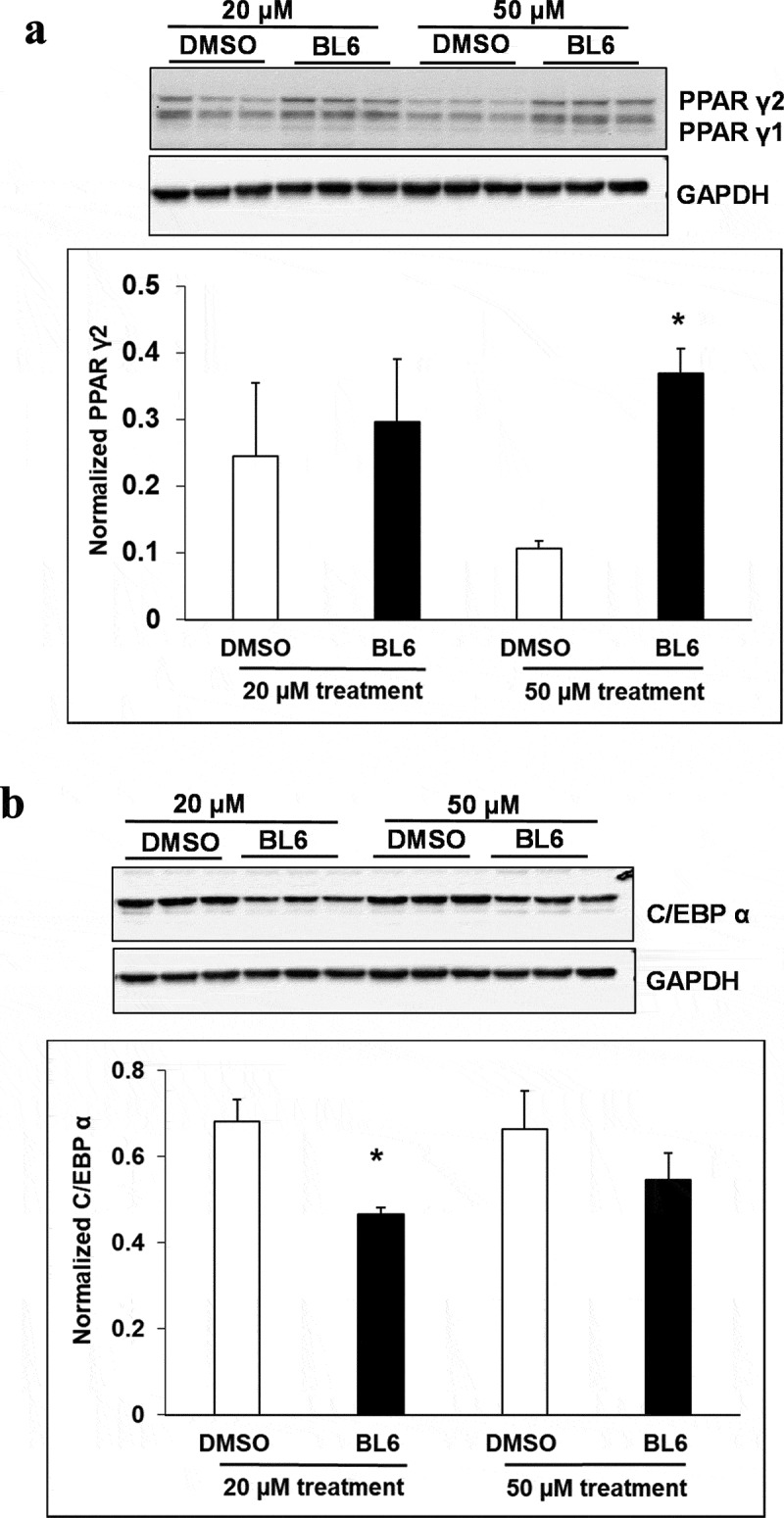
Protein expression of PPARγ, C/EBP α, FASn, adiponectin, pAKT and Glut4 for 20 µM, and 50 µM of BL6 treatment along with DMSO control. Protein lysates from 3T3-L1 cells treated with 20 µM BL6, and 50 µM BL6 during differentiation were separated on a SDS-PAGE gel, transferred onto a nitrocellulose membrane and immunoblotted with PPARγ, C/EBPα, FASn, adiponectin, pAKT, Glut4 and GAPDH antibodies. Graphs show average density of protein bands normalized to GAPDH and Akt. The effect of each dose of BL6 was compared to its own DMSO control. Data are presented as average ± sd (n = 3). Statistical significance was determined by Student TTEST (P < 0.05) and shown by asterisks on top of the bar.

**Figure 4.**
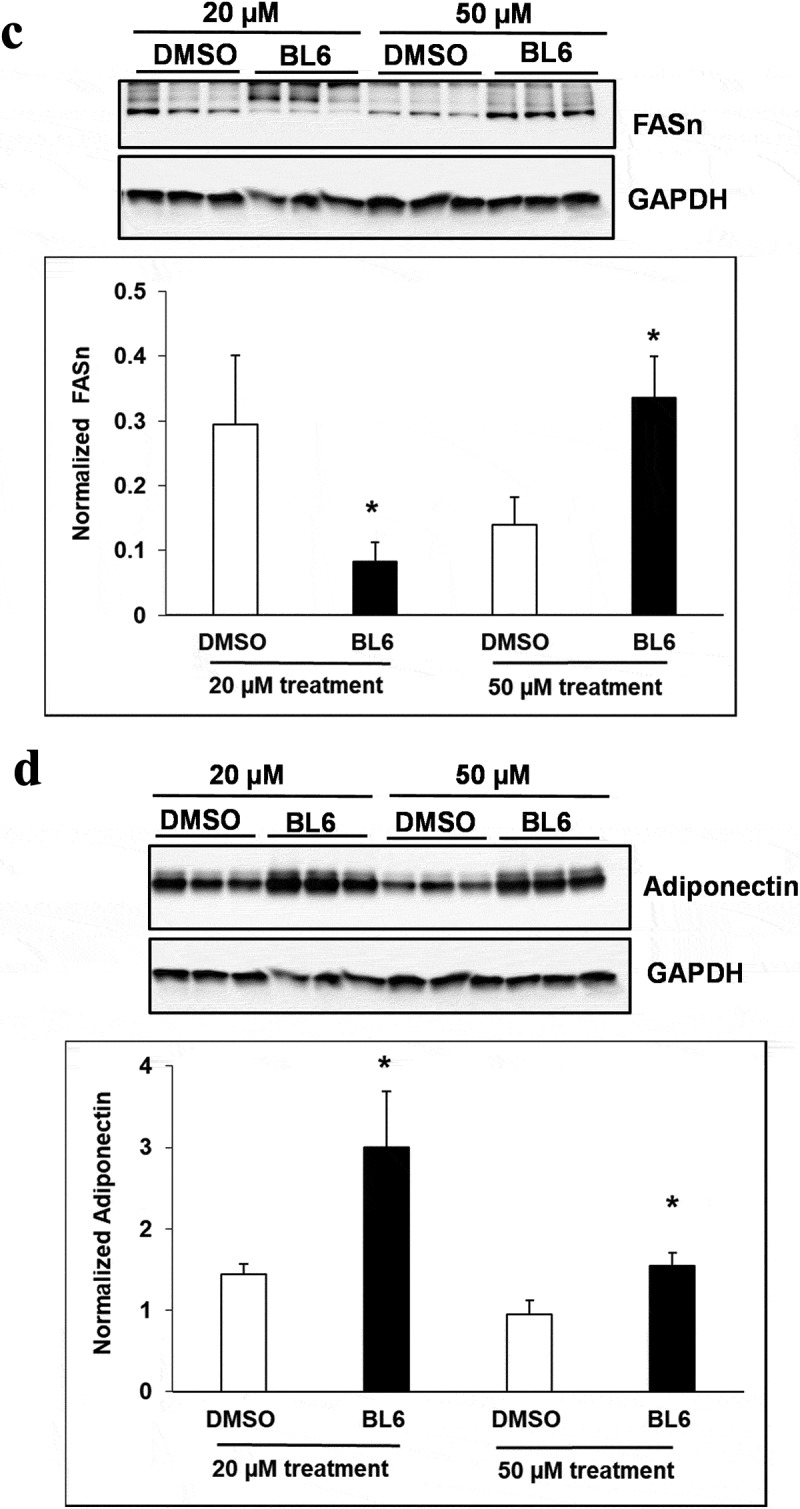
Continued

**Figure 4.**
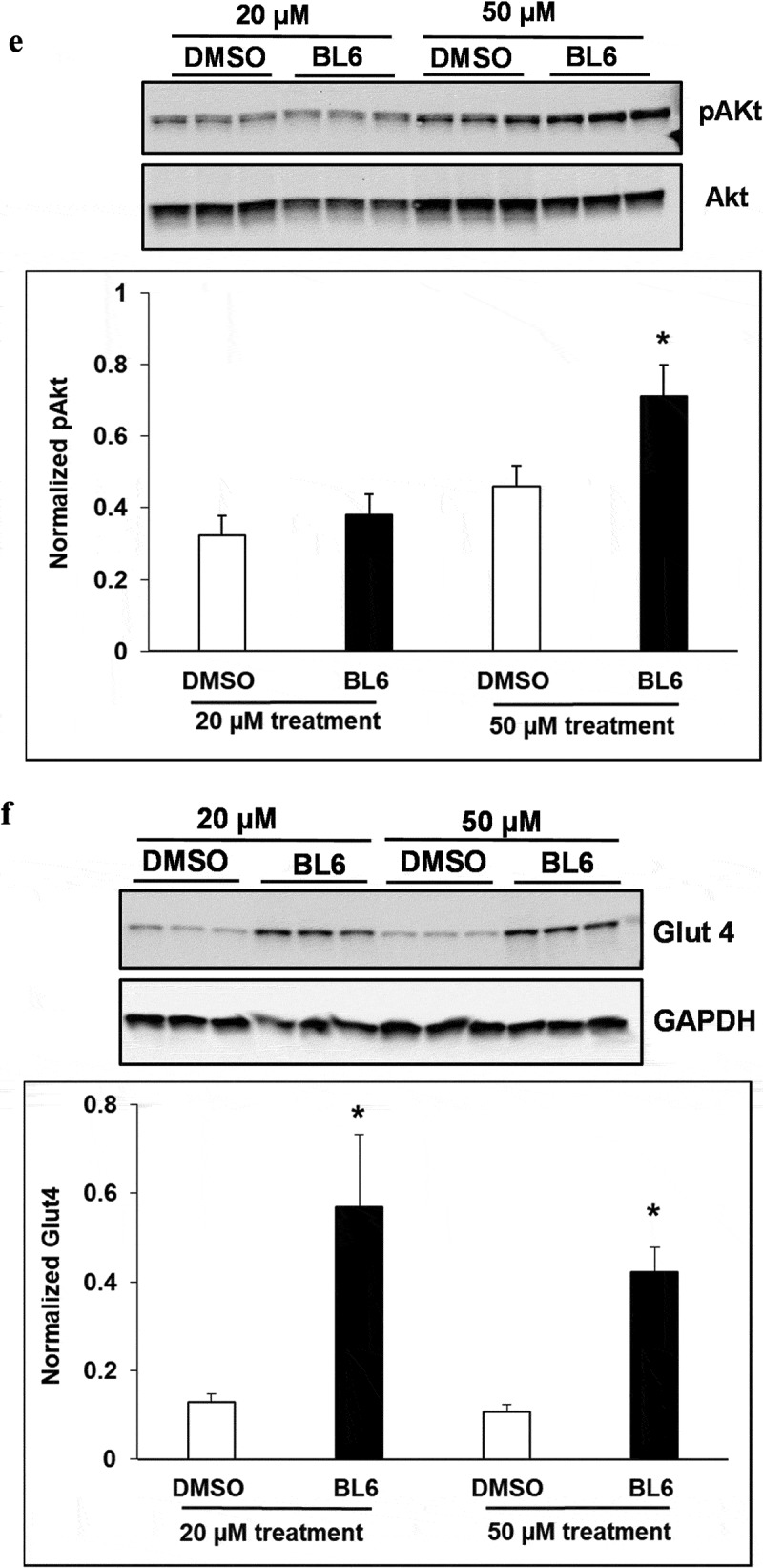
Continued

**Figure 5.**
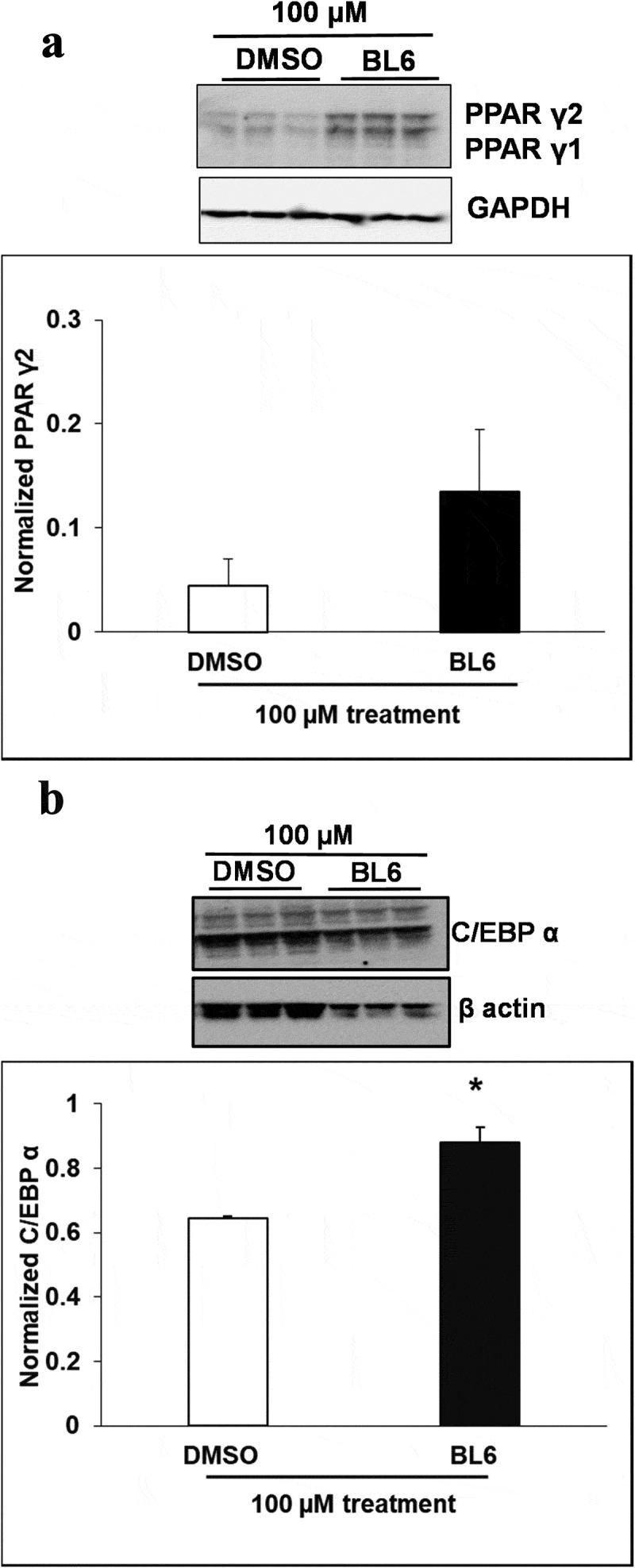
Protein expression of PPARγ, C/EBP α, FASn, adiponectin, pAKT and Glut4 for 100 µM of BL6 treatment along with DMSO control. Protein lysates from 3T3-L1 cells treated with 100 µM BL6 during differentiation were separated on a SDS-PAGE gel, transferred onto a nitrocellulose membrane and immunoblotted with PPARγ, C/EBP α, FAS, adiponectin, pAKT, Glut4, β actin, α tubulin, and GAPDH antibodies. Graphs show average density of protein bands normalized to either GAPDH, β actin or α tubulin. The effect of 100µM BL6 on protein expression was compared to its own DMSO control. Data are presented as average ± sd (n = 3). Statistical significance was determined by Student TTEST (P < 0.05) and shown by asterisks on top of the bar. The experiment was repeated a minimum of three times.

**Figure 5.**
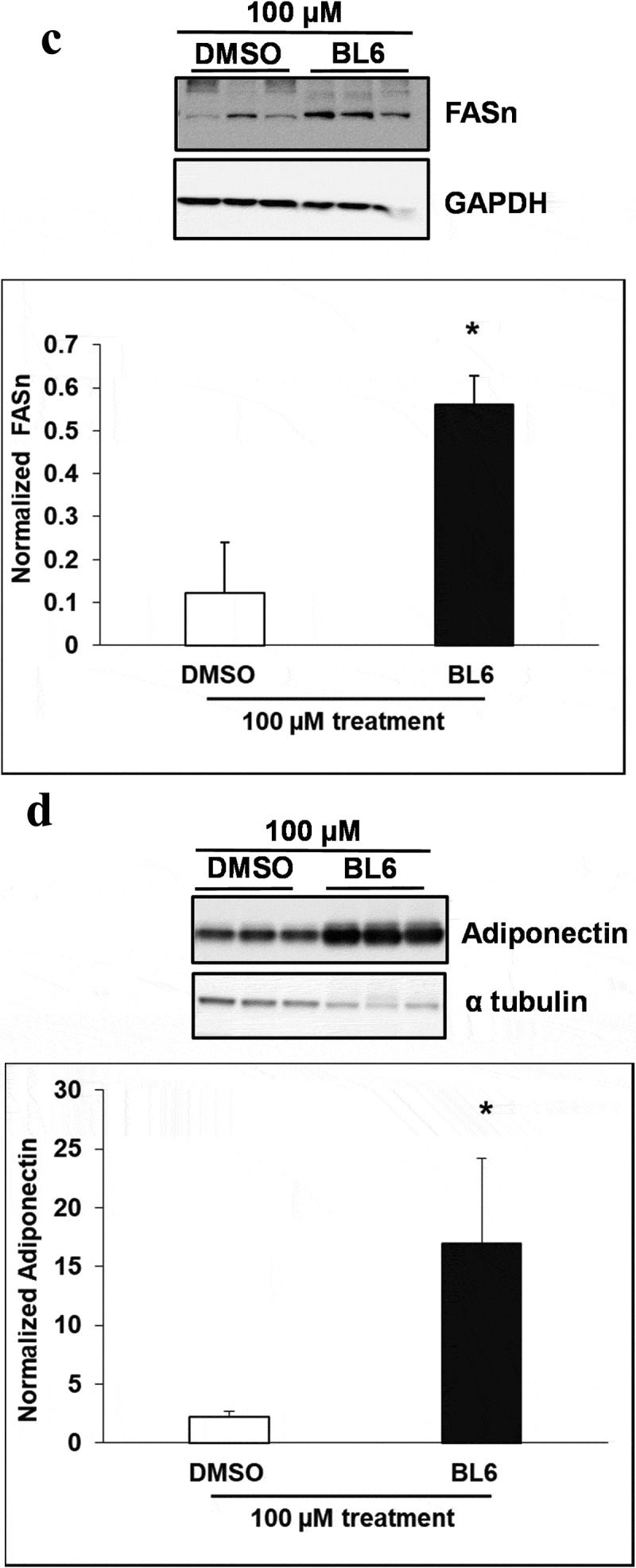
Continued

**Figure 5.**
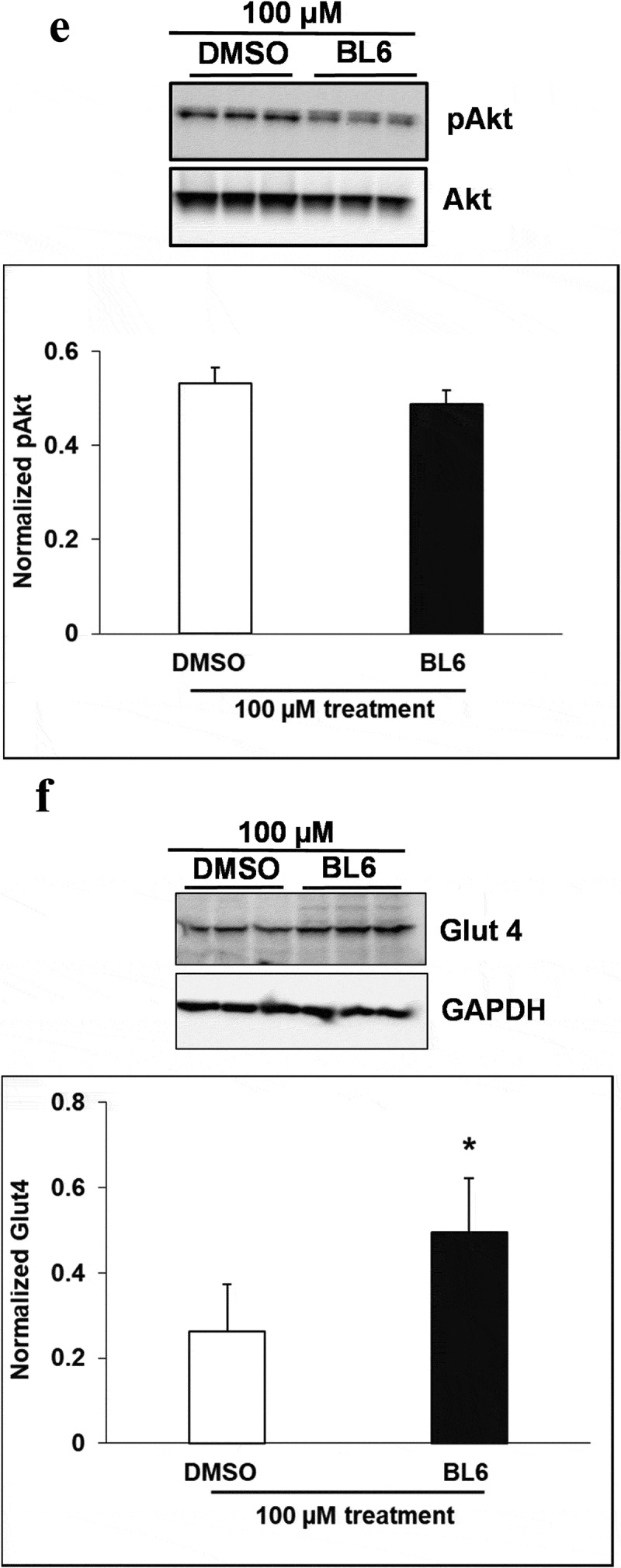
Continued

### Experiment 4: BL6 enhances cellular glucose uptake independent of adipogenesis

**Figure 6.**
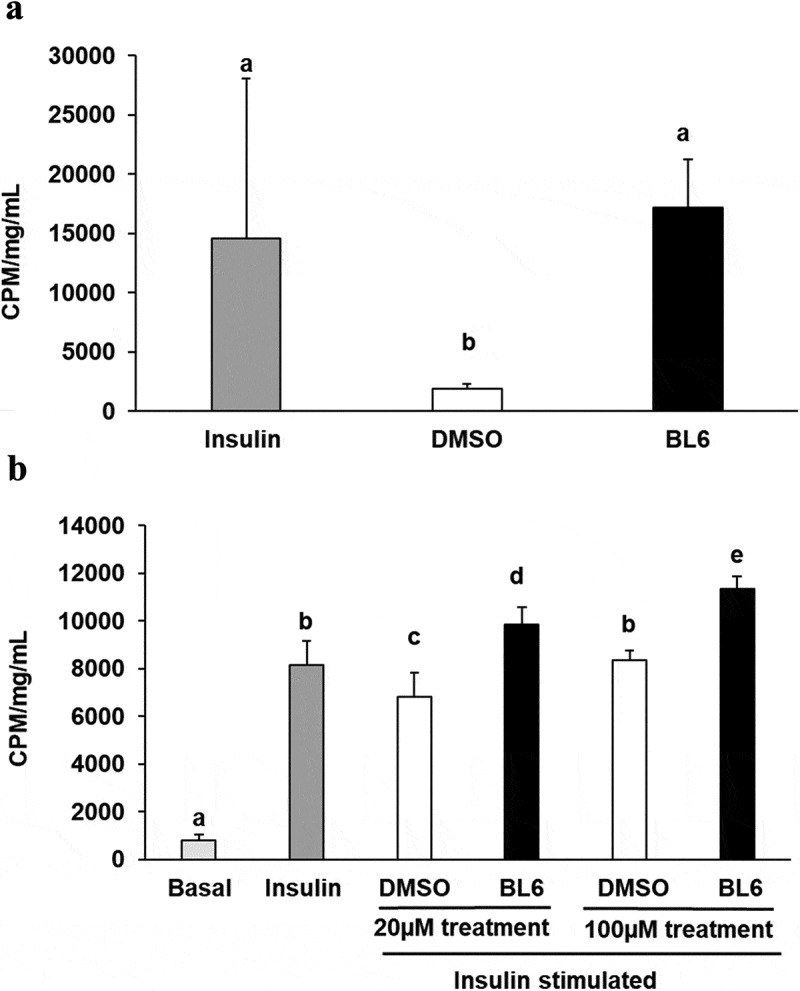
Glucose uptake for 20 µM and 100 µM of BL6 treated adipocyte cells. 3T3-L1 pre-adipocyte cells were treated with 20 µM, and 100 µM of BL6 along with MDI for 8 days during differentiation. Glucose uptake assay was performed following differentiation. (a) Insulin independent glucose uptake was determined with 100µM BL6 treated cells. Y-axis represents glucose uptake in CPM/mg/mL. (b) Similarly the effect of insulin during block to adipogenesis when cells were treated with 20µM and 100 µM of BL6 was determined under insulin stimulated conditions. Basal group on the X-axis represents glucose uptake independent of insulin. Radioactive glucose uptake was normalized to total protein content. Data are presented as average ± sd (n = 6). Groups sharing different alphabet denotes statistical significance (P < 0.05) as determined by ANOVA. The experiment was repeated a minimum of three times.

To determine glucose metabolism following a reduction in adipogenesis, murine 3T3-L1 cells were treated with 0 µM, 20 µM or 100 µM BL6 during differentiation with MDI. Cells were differentiated for 8 days followed by glucose uptake assay. We first determined insulin independent effect of BL6 on cellular glucose uptake in cells treated with 100 µM dose during differentiation. As seen in Figure 6(a), compared to volume-adjusted DMSO treated control cells, BL6-treated cells significantly (p < 0.05) increase glucose uptake and similar levels to insulin-treated cells (Figure 6(a)). Next, we determined if block to adipogenesis affects insulin-stimulated glucose uptake. BL6 treatment dose-dependently increased glucose uptake compared with DMSO treated control cells and insulin-stimulated control cells (Figure 6(b), p < 0.05 as determined by ANOVA). The basal group represents glucose uptake by control adipocytes, whereas Insulin stimulation shows approximately a 14-fold higher glucose uptake in adipocytes (second bar from the left, Figure 6(b)). Increase in glucose uptake observed in treated cells suggests that despite the reduction in adipogenesis by BL6, these cells are metabolically healthy and improve glucose metabolism.

## Discussion

Obesity is a serious chronic disease that is linked with excess accumulation of adipose tissue and consequential metabolic and other health disorders, including insulin resistance, impaired glycemic control or diabetes. The expansion of adipose tissue is accompanied by the development of vasculature, as adipogenesis is tightly associated with angiogenesis [28]. Adipose tissue produces and secretes many different types of pro- and anti-angiogenic factors [29]. Several *in vivo* studies have proposed that inhibition of the development of the vascular network in adipose tissue may establish an anti-obesity therapy [14,16,29–31]. Angiogenesis inhibitors fumagillin and its chemical analog TNP-470 impair adipose tissue growth in mice by selectively inhibiting endothelial cell growth via suppression of methionine aminopeptidase [14,16]. On the other hand, some animal models suggest that restricting adipose tissue expansion may have a deleterious effect on glucose metabolism. Therefore, it would be important to consider glucose disposal, when angiogenesis inhibitors are tested as inhibitors of adipogenesis. It would be highly desirable if an angiogenic inhibitor reduces adipogenesis without reducing glucose uptake by adipose tissue.

In the current *in vitro* study, we evaluated the anti-angiogenic and anti-adipogenic property of a boron-based chemical compound BL6, for its potential development as an anti-obesity drug. We examined the effect of BL6 as an angiogenic inhibitor in HUVECs during tube formation. We also investigated the effect of BL6 on adipogenesis *in vitro* during differentiation of 3T3-L1 pre-adipocytes and regulation of adipogenic protein and gene expression. Glucose uptake by BL6-treated 3T3-L1 adipocytes was further determined by glucose uptake assay.

BL6 dose-dependently reduced angiogenesis in HUVECs (Figure 1) and adipogenesis in 3T3-L1 cells (Figure 2). In 3T3-L1 cells, BL6 significantly suppressed differentiation from pre-adipocytes to adipocytes as determined by oil Red O staining, which specifically stains triglycerides proportional to cell differentiation or lipid accumulation [24]. We did not observe any difference in lipid accumulation with 20µM BL6 compared with its DMSO treated control, but both 50 µM and 100 µM concentration of BL6 suppressed lipid accumulation significantly (P < 0.05)(Figure 2(a,b)). In contrast, a previous *in vitro* study demonstrated higher differentiation rate of pre-adipocytes to adipocytes when treated with the natural angiogenic inhibitor, fumagillin [32]. However, fumagillin and its analog derivative TNP-470, have been shown to have inconsistent anti-obesity effects *in vivo* [33,34]. Thus, there appears to be differences with the anti-adipogenic potential of fumagillin and its derivative [14]. Oil Red O stained microscopic images also showed smaller and fragmented lipid droplets within adipocytes treated with 100 µM of BL6 compared with control. This suggests that compound BL6 may also affect the formation of the structural protein coating lipid droplets, such as perilipin [35].

BL6 reduces adiponectin gene expression in 3T3-L1 adipocytes, which supports the lower adipogenic differentiation or lipid accumulation observed. However, in the presence of BL6, protein translation for adiponectin was higher compared to control cells. BL6 suppresses PPARγ gene expression, the master regulator of adipocyte differentiation, at the higher 100 µM dose, but not with 20 µM or 50 µM dose. SREBP1, an upstream molecule of PPARγ, which regulates PPARγ expression and FAS a molecule downstream from PPARγ are both significantly down-regulated by treatment with 100 µM BL6. This suggests that BL6 suppresses PPARγ directly or through down-regulation of SREBP1 and affects adipocyte differentiation via downregulation of FAS expression. Reduced level of FAS expression is consistent with our oil Red O data showing less lipid accumulation.

Interestingly, BL6 did not significantly influence the C/EBP β gene expression. C/EBP β is an early regulator of adipocyte differentiation and we collected protein and RNA following 8 days of cellular differentiation, this might explain the lack of observable change with C/EBP β expression by day 8 [36].

C/EBPα, a member of the self-regulatory loop with PPARγ, is also significantly suppressed by 100µM BL6. Despite the reduction in adipogenesis, BL6 significantly increases cellular glucose uptake in 3T3-L1 cells in a dose-dependent manner (Figure 6). C/EBPα improves insulin sensitivity by upregulating insulin receptor (IR) insulin receptor substrate-1 (IRS-1) and Glut4 [37] and Wu Z et al. suggested that PPARγ alone can induce Glut 4 in absence of C/EBPα [38]. Therefore, the non-significant effect of BL6 on PPARγ expression may positively affect glucose uptake in these cells. This suggests improvement in glucose metabolism despite impaired adipogenesis and adiponectin secretion, which would be important for future in vivo studies and developing BL6 for therapeutic use.

DMSO treatment itself inhibits the expression of adipocyte phenotype and expression of adipogenic genes [39], which may affect our ability to observe significant differences at lower concentrations of BL6. To capture the true effect of compound BL6, we have compared the different BL6 dose-treated cells to its respective DMSO volume control and measured statistically differences between the two groups.

Interestingly, despite a significant reduction in adipogenic gene expression, we observed a contradictory increase in protein translation (Figures 4 and 5). The increased protein translation for pAKT, Glut4 and adiponectin support concurrent dose-dependent increase in cellular glucose uptake in these cells. The results obtained are not in error as independent experiments have shown similar results, but the observed dichotomy between RNA and protein expressions for the same gene could be due to several reasons, including an effect on protein ubiquitination, that would prevent protein degradation or protein stability may be increased due to post-translational modifications like phosphorylation, acetylatylation, and glycosylation, or the protein may be a long-lived protein which gets accumulated over time while the mRNA turnover is quick [40].

In conclusion, inhibition of MetAP2, a regulatory element for angiogenesis and a target molecule for anti-angiogenic compounds are a promising approach to treating diabetes, obesity, and associated metabolic disorders. Pre-clinical and clinical studies have shown, inhibition of fat mass expansion and improved glycemic control by MetAP2 inhibitors [14–20]. These findings suggest a possible therapeutic intervention of obesity and obesity-associated disorders by targeting the vascular compartment. However, a long-term phase 2 clinical trial with beloranib, a MetAP2 inhibitor was associated with venous thrombotic adverse events likely resulting from drug effects on vascular endothelial cells (ECs) [21]. Promising findings observed with various studies support further investigation of other MetAP2 inhibitors that might alleviate some of these risks and adverse effects previously observed. ZGN-1061, a MetAP2 inhibitor with improved safety profile and anti-diabetic properties than beloranib in mice [41] is in clinical development. In this study, compound BL6 blocks angiogenesis and adipogenesis *in vitro* yet improve cellular glucose uptake warranting future *in vivo* analysis of its safety and efficacy to develop as a possible anti-obesity therapeutic agent.
