# Supplementary material for: A MetAP2 inhibitor blocks adipogenesis, yet improves glucose uptake in cells
Source: Adipocyte. 2019 Jul 2;8(1):240–53. doi: 10.1080/21623945.2019.1636627 (PMC6768232; doi:10.1080/21623945.2019.1636627)
Supplement: Supplemental Material [file kadi-08-01-1636627-s001.docx]

**Supplemental Figure1.** (A) Chemical structure of MetAP2 inhibitors and BL#6. (B) Chemical structure for compound BL6

**A**

**B**

The chemical structure of fumagillin was completely reshaped (Supplemental Figure 1A), by changes at position **3, 4, 5 and 6.** The 6-position of fumagillin plays an important role in its activity. The parent compound at the position 6 contains an ester group, which is very prone to hydrolysis. To improve the efficacy and stability of the esters, we reshaped the molecules by introducing an exocyclic double bond at the position 6. We also introduced a 7 membered carbon atom in place of 9 membered open chain alkene spacers by constrained phenyl ring and kept the acid group intact. Our BL#6 compound contains a boron atom. We are exploiting the recent appreciation of boron chemistry. Boron containing pharmacophore groups ^1^ interact with a target protein not only through the hydrogen bonds but also through reversible covalent bonds. This produces potent biological activity (e.g. antifungal, antiparasitic, and protease inhibition), a concept that is well supported by literature ^1, 2^. BL6 contains boron based active moiety. Boron-containing compounds are currently in clinical use, such as the proteasome inhibitor, FDA approved Velcade (bortezomib) for multiple myeloma and the antifungal therapies, KERYDIN™ (Tavaborole, FDA approved in 2014), Crisaborole (Eucrisa, FDA approved in 2016) and antibacterial β-lactam β-lactamase inhibitor Vaborbactam (for complicated urinary tract infections and pyelonephritis-FDA approved 2017). BL6 is a new class of boron containing MetAP2 inhibitor, unique in nature and different in chemical structure with different pharmacophore group (Supplemental Figure 1B).

1. Das BC, Thapa P, Karki R, Schinke C, Das S, Kambhampati S *et al.* Boron chemicals in diagnosis and therapeutics. *Future Med Chem* 2013; **5**(6)**:** 653-76.

2. Zhao X, Xiaoli, Zong H, Abdulla A, Yang ES, Wang Q *et al.* Inhibition of SREBP transcriptional activity by a boron-containing compound improves lipid homeostasis in diet-induced obesity. *Diabetes* 2014; **63**(7)**:** 2464-73.
